# Descriptive analysis of the cases published in the Align^®^ Global Gallery

**DOI:** 10.1590/2177-6709.29.5.e242474.oar

**Published:** 2024-10-07

**Authors:** Luísa Schubach da Costa BARRETO, Rafael Cunha BITTENCOURT, Marcella Barreto FERREIRA, Sarah Braga Sayão de PAULA, Cláudia Trindade MATTOS, José Augusto Mendes MIGUEL

**Affiliations:** 1 Rio de Janeiro State University (UERJ), School of Dentistry, Department of Social and Preventive Dentistry (PRECOM) (Rio de Janeiro/RJ, Brazil).; 2 Federal Fluminense University (UFF), School of Dentistry, Department of Orthodontics (Niterói/RJ, Brazil).; 3 Federal University of Rio de Janeiro (UFRJ), School of Dentistry, Department of Pediatric Dentistry and Orthodontics (Rio de Janeiro/RJ, Brazil).; 4 University of Michigan, School of Dentistry, Department of Orthodontics and Pediatric Dentistry (Michigan, USA).; 5 Brazilian Board of Orthodontics and Facial Orthopedics, Department of Orthodontics (Brazil).

**Keywords:** Clinical protocols, Orthodontic appliances, removable, Orthodontics, Protocolos clínicos, Aparelhos ortodônticos removíveis, Ortodontia

## Abstract

**Introduction:**

Social media enhanced access to information, making it easier to share dental treatments.

**Objective:**

This study aimed to conduct a descriptive analysis of the clinical cases published on the Align^®^ Global Gallery platform.

**Material and Methods:**

A retrospective cross-sectional study of 1,582 cases was conducted, data extracted referred to the following basic information: case number; patient’s age; reported gender; Invisalign^®^ package modality; treatment time; aligner exchange protocol; total number of aligners per arch; type of retainers, and inclusion of initial and final panoramic and cephalometric radiographs.

**Results:**

The majority were young (mean age 24.6 years, SD = 11.6), female patients (69.1%) with Class I malocclusion (39.4%) and crowding (77.9%). Comprehensive treatment was common (66.5%), with an average treatment time of 18 months (SD = 8.56; 95% CI = 17.6-18.5), with the most frequently reported aligner exchange protocol being 7 days (49.5%), with an average of 50.6 aligners in the upper arch (SD = 26.9; 95% CI = 49.2-51.9), and 48.7 in the lower arch (SD = 26.1; 95% CI = 47.4-50.0). Arch expansion (66.9%) and interproximal reduction (59.7%) were common approaches, while extractions were rare (4.3%). In most cases, initial lateral cephalometric (80.4%) and panoramic (93.3%) radiographs were presented. However, the final radiograph count dropped, with lateral cephalometric at 69.2%, and panoramic at 82.2% of cases.

**Conclusion:**

Cases in the Align^®^Global Gallery mostly feature Class I patients with crowded teeth, treated with expansion and interproximal reduction. The absence of standardized information and post-treatment data restricts the applicability of these findings to broader Invisalign^®^ treatment trends.

## RESUMO

**Introdução:**

As mídias sociais melhoraram o acesso às informações, facilitando o compartilhamento de tratamentos odontológicos.

**Objetivo:**

O objetivo desse estudo foi realizar uma análise descritiva dos casos clínicos publicados na plataforma Galeria Global Align^®^.

**Métodos:**

Foi realizado um estudo transversal retrospectivo de 1.582 casos, coletando as seguintes informações básicas: número do caso; idade; sexo; modalidade do Invisalign^®^; tempo de tratamento; protocolo de troca; número total de alinhadores; contenções; radiografias panorâmicas e cefalométricas.

**Resultados:**

A maioria dos pacientes era jovem (idade média de 24,6 anos, DP = 11,6), do sexo feminino (69,1%), com má oclusão de Classe I (39,4%) e apinhamento (77,9%). O tratamento “Comprehensive” foi comum (66,5%), com um tempo médio de tratamento de 18 meses (DP = 8,56; IC 95% = 17,6-18,5), sendo que o protocolo de troca de alinhadores mais frequentemente relatado foi o de sete dias (49,5%), com uma média de 50,6 alinhadores na arcada superior (DP = 26,9; IC 95% = 49,2-51,9) e 48,7 na arcada inferior (DP = 26,1; IC 95% = 47,4-50,0). Expansão da arcada (66,9%) e a redução interproximal (59,7%) foram abordagens comuns, enquanto extrações foram raras (4,3%). Na maioria, foram apresentadas radiografias iniciais cefalométricas (80,4%) e panorâmicas (93,3%). No entanto, a contagem de radiografias finais caiu, cefalométrica lateral em 69,2% e a panorâmica em 82,2% dos casos.

**Conclusão:**

A Galeria Global Align^®^ apresenta principalmente pacientes de Classe I com dentes apinhados, tratados com expansão e redução interproximal. A ausência de informações padronizadas e de dados pós-tratamento restringe a aplicabilidade dessas descobertas a tendências mais amplas de tratamento Invisalign^®^.

## INTRODUCTION

Clear aligners have become a significant presence in modern orthodontics, offering notable advantages over traditional methods.^1^ Their transparent and removable design provides a more aesthetically pleasing, hygienic, and comfortable treatment option, particularly appealing to adults who prioritize discreetness. Developed since 1999 by Align Technology^®^,^2^ these aligners gradually correct tooth alignment through digital planning, though their efficacy in complex cases remains controversial.

The rise of social media and increased access to information has facilitated the sharing of successful dental treatments, including through platforms like the Align^®^ Global Gallery. This online resource, currently featuring around 1,900 cases, allows professionals to showcase their outcomes, attracting over 200 thousand annual views from 160 countries. Professionals who wish to publish their cases must send a legal documentation consisting of photographs, radiographs and the digital planning of the case (ClinCheck^®^). Despite its popularity, it is uncertain whether the gallery offers a comprehensive overview of treatment trends worldwide, given the variability in diagnoses and treatment plans.

The primary audience of the gallery likely comprises practitioners actively engaged in or aspiring to offer Invisalign^®^ treatments, seeking insights into successful cases for their own patients. Therefore, the aim of the present study was to carry out a descriptive analysis of the clinical cases published in the Align^®^Global Gallery.

## MATERIAL AND METHODS

This study analyzed 1,582 cases from the Align^®^ Global Gallery, accessed via <*https://alignglobalgallery.com*>. Data collection occurred from December 15, 2022, to January 4, 2023, including all cases publicly available in the Align^®^ Global Gallery during the flagged period, with two orthodontists and a scientific initiation scholarship student manually tabulating the information team (LSCB, RCB, and MBF, respectively), duly calibrated for the particularities of the collection. Basic patient demographics, treatment details, clinical diagnoses, and treatment modalities were collected from publicly available records. If any of this information was missing from the record, it was excluded. This exclusion criterion was applied due to the unavailability of complete data in the records. Only the available data was included. If there was any doubt in the collection of data, all researchers were called to a consensus meeting. Descriptive statistics and frequency tables were generated using Microsoft Excel (version 19) and Jamovi (version 1.6) softwares. 

## RESULTS

Among the parameters analyzed, a higher proportion of women (69.1%) sought treatment, with an average age of 24.6 years (SD = 11.6; 95% CI = 24.0-25.2). The Comprehensive treatment modality was most common (66.5%), followed by Invisalign^®^ First (21.1%), suitable for mixed dentition. Treatment duration averaged 18 months (SD = 8.56; 95% CI = 17.6-18.5), with a predominant aligner exchange protocol of 7 days (49.5%). The upper arch typically required 50.6 aligners (SD = 26.9; 95% CI = 49.2-51.9), and the lower arch, 48.7 aligners (SD = 26.1; 95% CI = 47.4-50.0) (Table 1).

**Table 1:** Descriptive data regarding the general information of the cases.

Parameter	Mean (SD; 95% CI)
Age (years)	24.6 (11.6; 24.0-25.2)
Time (months)	18 (8.56; 17.6-18.5)
Number of aligners	
Upper arch	50.6 (26.9; 49.2-51.9)
Lower arch	48.7 (26.1; 47.4-50.0)
Parameter	Percentage (%)
Retainers in upper arch	
Fixed	28.3%
Vivera	26.2%
Hawley	8.4%
Aligners	3.6%
Other	33.3%
Retainers in lower arch	
Fixed	23.7%
Vivera	28.2%
Hawley	8.3%
Aligners	3.7%
Other	35.8%
Protocol (days)	
7	49.5%
10	22.8%
15	24.0%
Exams	
Initial panoramic	93.3%
Initial cephalometric	80.4%
Final panoramic	82.2%
Final cephalometric	68.2%
Package	
Comprehensive	66.5%
First	21.1%
Lite	7.8%
Moderate	0.6%
Express	0.1%
Other	3.9%
Sex	
Female	69.1%
Male	30.9%

SD = standard deviation; 95% CI = 95% confidence interval.

**Figure 1:**
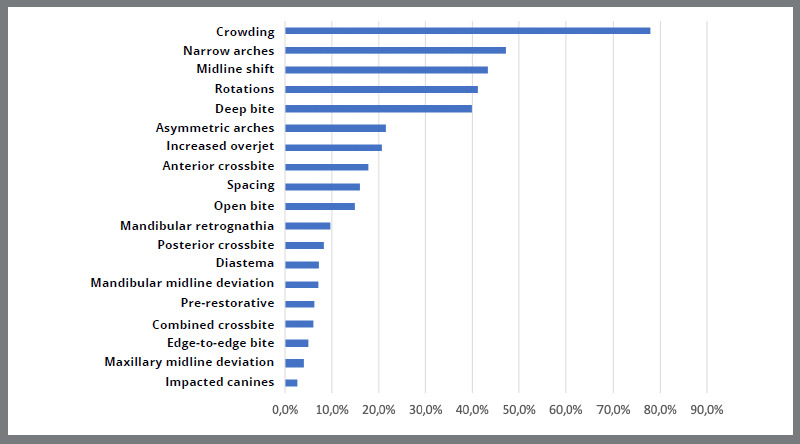
Descriptive data on the types of malocclusion treated.

Malocclusion classification revealed Angle Class I as most prevalent (39.4%), followed by Class II division 1 (30.0%), Class III (14.7%), and Class II division 2 (11.4%). Dental crowding was the predominant malocclusion type (77.9%), followed by narrow arches (47.3%), midline deviation (47.3%), rotations (41.2%), and deep bite (39.9%). Impacted canines were the least common (2.7%) (Fig 1). 

**Figure 2:**
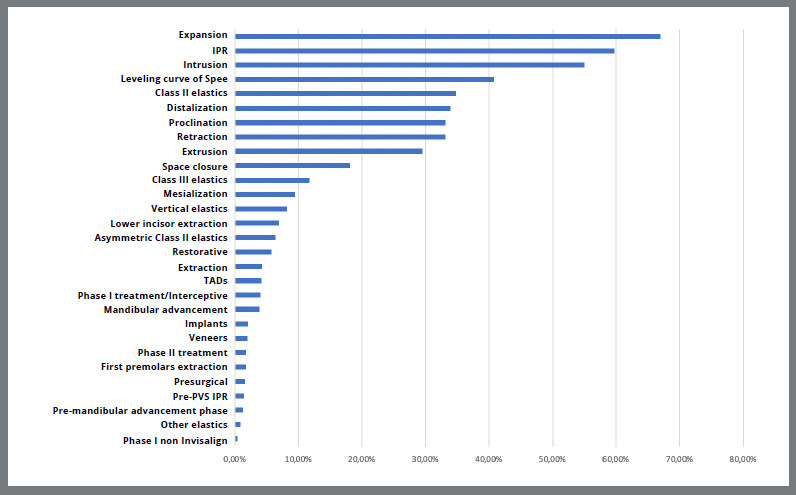
Descriptive data on the types of treatments performed.

Treatment approaches included arch expansion (66.9%), interproximal reduction (IPR) (59.7%), intrusive movements (55.0%), and leveling of the curve of Spee (40.8%). The precise amount of IPR in millimeters is not provided in the Global Gallery. Extraction practices were notably underreported, with an overall extraction rate of 4.3%. First premolar extraction accounted for 1.8%, while lower incisor extraction was slightly higher at 6.9% (Fig 2).

Retainer types varied (Table 1), with the “other” category being most frequent (33.3% upper, 35.6% lower), followed by Vivera^®^ retainers (26.2% upper, 28.2% lower) and fixed retention (28.3% upper, 23.7% lower). Hawley-type retainers were less common (8.4% upper, 8.3% lower), followed by retainers made with another aligner type (3.6% upper, 3.7% lower). Most cases included initial lateral cephalometric (80.4%) and panoramic (93.3%) radiographs, but the number of final radiographs decreased (69.2% lateral cephalometric, 82.2% panoramic).

## DISCUSSION

Malocclusions impact quality of life, affecting physical, social, and psychological aspects if untreated.^3^ Satisfaction and quality of life post-Invisalign^®^ treatment are linked to improved aesthetics and nutrition.^4^ Positive professional-patient relationships during treatment are crucial for satisfaction. The Align^®^ Global Gallery showcases successful cases, but it doesn’t fully represent device performance or treatment demand. For example, the prevalence of Class I and crowding cases, and the scarcity of extraction cases should not imply exclusive Invisalign^®^ use for these scenarios. Gallery case selection may be influenced by practical factors, and may not encompass the full spectrum of viable treatment options supported by scientific literature, including peer-reviewed articles and research.

To submit a case to the Align^®^ Global Gallery, both the professional and the patient sign image use consent forms. The professional provides treatment data, approved by the company, with numerical data requiring at least two additional aligner orders. Submission includes ClinCheck^®^, initial and final intraoral photographs, and optional radiographs, with cases needing approval before publication. These results serve as a company advertisement, showcasing treatment possibilities, but representing only a portion of suitable outcomes for public viewing.

The Align^®^ Global Gallery displays approved cases, but studying unpublished ones is crucial. The Global Gallery does not make publicly available the date of inclusion of each treatment, nor the year or period of beginning and end of the orthodontic intervention performed, preventing comparison between techniques used in different generations of Invisalign^®^ aligners and attachments. The present research found that young women, averaging 24 years old, were the most common patients, aligning with the study by Meier et al.^5^ They suggested women aged 20 to 29 primarily seek Invisalign^®^ for aesthetics, accepting 18 to 30 months of treatment. The present findings corroborate this, indicating an average treatment duration of 18 months, which may be considered extended, especially given that most cases are Class I malocclusions. This extended duration could be attributed to several factors, including patient adherence, the interval between aligner changes, the extent of crowding, among others.

The duration of treatment shows a strong correlation with the number of additional aligners needed to achieve desired results, as well as with the number of aligners required, regardless of the phases involved.^6^ In essence, a higher number of aligner sets usually means a longer treatment duration. In the present study, an average of 46 upper and 44 lower aligners was observed, with a standard aligner exchange protocol of seven days. However, data on the number of additional aligners needed to complete cases was unavailable, hindering the comparison between aligners used and additional aligners required. We recommend incorporating a section in the Align^®^ Global Gallery that offers detailed information, including the quantities of additional aligners used to achieve the desired treatment outcomes. Therefore, this scenario does not represent the exact number of aligner changes, as patients may not switch aligners precisely every seven days. Some patients might lose an aligner, experience a poor fit, reuse a previous aligner, or use one aligner for an extended period. Additionally, the number of additional aligners can vary, and the process can be delayed due to waiting for a second kit, which involves scanning, planning and the arrival of the new aligners.

During Invisalign^®^ treatment, multiple phases are often necessary,^6^ as aligners typically deliver only about 50% of the planned movements.^7^ On average, each treatment requires at least two scans for additional aligners, to plan and the produce new aligner sets.^2^ It’s essential to note that we might be overestimating the treatment’s efficiency and predictability. Therefore, patients should be advised that multiple treatment phases are usually needed, and orthodontic appliances with hybrid mechanics may be necessary.^2^

The Comprehensive treatment modality was the most commonly reported. This approach is designed for complex cases, offering an unlimited number of aligners over an extended period (five years). These cases, showcased in the Global Gallery^®^, are considered successful and often attract attention, promoting the Invisalign^®^ brand by demonstrating its effectiveness. This could explain why more than half of the published cases fall into this treatment modality.

A robust method for classifying malocclusion is crucial for accurately recording its prevalence across different populations, enabling comparisons between various groups.^8^ Among the cases treated, Angle Class I malocclusion was the most prevalent, followed by Class II division 1, Class III, and Class II division 2. These findings are supported by Lombardo et al.^9^ review, which found that two-thirds of the global population have Angle Class I malocclusion, followed by Class II and Class III, respectively. This pattern aligns with the average treatment time in the present study, as Class I cases usually involve fewer anteroposterior movements, leading to less complex cases and shorter treatment times.

Dental crowding was significantly the most common type of malocclusion in the present study. It is known that dental crowding, besides being highly prevalent, often develops later in the evolution of occlusion, due to changes in arch dimensions, even in individuals with untreated normal occlusion.^10^ ^,^ ^11^ Additionally, anterior crowding is notably perceived as the most unattractive malocclusion, both compared to other malocclusions^12^ ^,^ ^13^ and facial features.^14^ ^,^ ^15^ This perception, along with its prevalence, likely drives individuals to seek solutions for its resolution.

In addition to the midline deviation, the second most reported malocclusion was the presence of narrow arches. Coincidentally, arch expansion was the most used approach during treatments. This makes sense when we analyze the most reported malocclusions, such as dental crowding and narrow arches, in which expansion promotes space gain by increasing the perimeter of the arches. However, on average, the amount of expansion predicted with the Invisalign^®^ system is not achieved, especially in second molars. They are often the terminal teeth and have shorter clinical crowns, further limiting the action of the aligners.^16^ On the other hand, a systematic review conducted by Bouchant et al.^17^ found that maxillary expansions with Invisalign^®^ appear to be possible, when overcorrected in the ClinCheck^®^, but in a dentoalveolar way, through the buccal inclination of the posterior teeth.

Regarding the predictability of dentoalveolar expansions, it also is important to discuss their long-term stability. It seems to present satisfactory stability, except for the inter-premolar region, which tends to a significant recurrence after completion of treatment,^18^ ^-^ ^20^ mainly during the first-year post-treatment.^19^ Consideration must be given to the regime and type of retainer,^20^ as well as the amount of expansion carried out. In a recent study,^21^ mild levels of upper arch expansion achieved with Invisalign^®^ in adult patients did not result in any significant loss of alveolar bone thickness. However, expansions of approximately 3 mm in the region of the upper premolars led to a reduction in the buccal bone plate in the middle portion of the root of these elements.

Interproximal reduction (IPR) was the second most common treatment method, being especially useful for creating space in cases of dental crowding, the most prevalent malocclusion in this study. In a systematic review by Gómez-Aguirre et al.,^22^ no adverse effects on enamel demineralization, cavity incidence, periodontal changes, or tooth sensitivity were found after the IPR procedure. However, the actual amount of IPR performed on upper and lower teeth is often less than planned in ClinCheck^®^,^6^ potentially leading to increased refinement needs or additional mechanics like expansion. This cautious approach by professionals may be due to concerns about creating residual spaces.

Extractions were rarely chosen as a treatment approach, which can be explained by various reasons. Some may prefer arch expansion and interproximal reduction to make space. Adults might be reluctant to undergo extractions. Additionally, controlling root movement during canine and incisor retraction with clear aligners can be challenging. Studies indicate that upper incisor retraction after premolar extraction with Invisalign^®^ aligners may be less effective than with fixed appliances.^23^ In such cases, there may be more lingual crown tip movement and an increase in overbite, leading to longer treatment times.^23^ Conversely, extraction of a lower incisor, when appropriately indicated and planned, can be a satisfactory treatment option.^24^

Orthodontic retainers are crucial post-treatment, ensuring stability in the teeth’s new position and preserving function, aesthetics, and the balance of the stomatognathic system.^25^ Removable plastic devices have gained popularity for their capability to encapsulate and maintain teeth in position.^26^ The present research found that the most frequently used retainers fell into the “other” category, probably including thermo plasticized retainers apart from Vivera^®^. Fixed retainers may present hygiene challenges, but with consistent periodontal care and thorough oral hygiene guidance, maintaining periodontal health is achievable.^27^ For significant expansions, it’s beneficial to utilize retainers involving all teeth in the arch and palate, like removable retainers with an acrylic plate, for about six months to prevent relapse.^28^ “Hawley” type retainers, maintaining free occlusal surfaces, provide better anterior and posterior occlusal contacts, compared to vacuum-formed retainers,^25^ ^,^ ^29^ favoring the physiological occlusal adjustment that occurs post-treatment.

A limitation of this study is the lack of standardized data, as professionals choose which diagnostic and treatment items to include, potentially introducing bias from diagnostic errors or omitted data. Additionally, the absence of post-treatment examinations in some cases makes it difficult to compare bone and root aspects. The anonymity of the panel judging the cases is another important consideration, as their identities, qualifications, and acceptance criteria are unknown.

When undertaking a comparative analysis of outcomes derived from cases treated with aligners,^30^ ^,^ ^31^ it is imperative to acknowledge that, despite the diversity of case types and professional testimonials cited within the literature, a predominant proportion of the cases may exhibit a lesser degree of complexity. A recent systematic review^32^ highlighted an abundance of articles focusing on dental expansion and crowding correction, potentially indicating a lack of documentation for extraction cases. It’s important to note that extraction cases are less common in publications, but their absence does not reflect treatment quality or effectiveness. A thorough approach is vital when assessing trends and outcomes in Invisalign^®^ published cases.

## CONCLUSION

The majority of cases in the Align^®^ Global Gallery feature Class I patients with dental crowding, typically treated with expansion and interproximal reduction. However, information about the most commonly used type of retainer and post-treatment stability is lacking. Due to the lack of standardized information and omitted data, it is challenging to conclusively state that the published cases represent a comprehensive global profile of Invisalign^®^ treatment trends.
